# Phenotypic and genotypic characterization of *Salmonella* Typhimurium isolates from humans and foods in Brazil

**DOI:** 10.1371/journal.pone.0237886

**Published:** 2020-08-18

**Authors:** Amanda Aparecida Seribelli, Marcelo Ferreira Cruz, Felipe Pinheiro Vilela, Miliane Rodrigues Frazão, Mario H. Paziani, Fernanda Almeida, Marta Inês Cazentini Medeiros, Dália dos Prazeres Rodrigues, Marcia R. von Zeska Kress, Marc W. Allard, Juliana Pfrimer Falcão

**Affiliations:** 1 Departamento de Análises Clínicas, Toxicológicas e Bromatológicas, Faculdade de Ciências Farmacêuticas de Ribeirão Preto–Universidade de São Paulo—USP, Brazil; 2 Instituto Adolfo Lutz de Ribeirão Preto, São Paulo, Brazil; 3 Fundação Oswaldo Cruz–FIOCRUZ, Rio de Janeiro, Brazil; 4 Food and Drug Administration—FDA, College Park, Maryland, United States of America; Cornell University, UNITED STATES

## Abstract

*Salmonella enterica* subsp. *enterica* serovar Typhimurium (*S*. Typhimurium) causes gastroenteritis in many countries. However, in Brazil there are few studies that have conducted a virulence characterization of this serovar. The aim of this study was to evaluate the virulence potential of *S*. Typhimurium strains isolated in Brazil. Forty *S*. Typhimurium strains isolated from humans (n = 20) and food (n = 20) from Brazil were studied regarding their invasion and survival in human epithelial cells (Caco-2) and macrophages (U937). Their virulence potential was determined using the *Galleria mellonella* larvae model combined with the analysis of virulence genes by whole genome sequencing (WGS). A total of 67.5% of the *S*. Typhimurium studied (32.5% isolated from humans and 35% isolated from food) invaded Caco-2 epithelial cells at levels similar to or greater than the *S*. Typhimurium SL1344 prototype strain. In addition, 37.5% of the studied strains (25% isolated from humans and 12.5% isolated from food) survived in U937 human macrophages at levels similar to or greater than SL1344. *S*. Typhimurium strains isolated from humans (40%) and food (25%) showed high or intermediate virulence in *G*. *mellonella* larvae after seven days exposure. Approximately, 153 virulence genes of chromosomal and plasmidial origin were detected in the strains studied. In conclusion, the ability of the *S*. Typhimurium to invade Caco-2 epithelial cells was strain dependent and was not related to the source or the year of isolation. However, *S*. Typhimurium strains isolated from humans showed greater survival rates in U937 human macrophages, and presented higher proportion of isolates with a virulent profile in *G*. *mellonella* in comparison to strains isolated from food suggesting that this difference may be related to the higher frequency of human isolates which contained plasmid genes, such as *spvABCDR* operon, *pefABCD* operon, *rck* and *mig-5*.

## Introduction

*Salmonella* Typhimurium has been an important cause of gastroenteritis in different parts of the globe [1–3]. It is important to emphasize that the transmission of this bacterium is mainly due to the ingestion of contaminated food such as eggs, beef, poultry, swine, and vegetables [4]. In addition, the transmission can occur person-to-person by fecal oral routes and, contaminated asymptomatic pets also can transmit to humans [4].

According to the Centers for Disease Control and Prevention (CDC) (2020), it was estimated that 1.35 million infections, 26,500 hospitalizations and 420 deaths occur in the United States every year due to salmonellosis [5]. In humans the symptoms are diarrhea, fever, and stomach cramps, with food being the main source of transmission of this disease. Therefore for prevention and control of *Salmonella* spp. food safety and hygenic handling practices are very important [5].

In Brazil, *Salmonella* spp. has been one of the main bacterial genera isolated from foodborne outbreaks [6]. However, until now there are few published studies that have characterized the possible differences between Brazilian *S*. Typhimurium strains isolated from human and food sources. No studies have examined the invasiveness of these isolates to Caco-2 epithelial cells (human colon adenocarcinoma), their survival in U937 human macrophages, or described the repertoire of virulence genes present by whole genome sequencing (WGS).

Several genes are responsible for the virulence of *Salmonella* spp. in different hosts. The Type III Secretion System (T3SS) is an important virulence factor for the invasion and survival of this pathogen in epithelial and phagocytic cells at the beginning of the infection [7, 8]. The genes are located mainly in two regions of the chromosome denominated pathogenicity islands 1 and 2 (SPI-1 and SPI-2) encode by T3SS [7, 8].

The virulence of *Salmonella* usually has been studied in mice, but there are some studies that used alternative infection models such as *Galleria mellonella* in which larvae are easily grown in large numbers and have components of the innate immune response similar to mammals, formed mainly by hemocytes and opsonins [9].

Whole genome sequencing is a powerful tool for assessing phylogenetic relationships, virulence, and antimicrobial resistance content, as well as providing information about the presence of plasmids, among other data in different bacterial genera [10–12].

The aims of this study were to evaluate *S*. Typhimurium isolates from humans and foods in Brazil and their ability to invade Caco-2 epithelial cells, the ability to survive in U937 human macrophages, and to assess virulence in the *Galleria mellonella* infection model, and lastly to characterize the repertoire of virulence genes present through WGS.

## Materials and methods

### Bacterial strains

A total of 40 *S*. Typhimurium strains isolated from humans (n = 20) and food (n = 20) between 1983 to 2013 in Brazil were studied (Table 1). These isolates were selected from the collections of the Adolfo Lutz Institute of Ribeirão Preto (IAL-RP) and of the Oswaldo Cruz Foundation from Rio de Janeiro (FIOCRUZ). *S*. Typhimurium SL1344 prototype strain was used as control in all experiments.

**Table 1:** Characteristics of the 40 *Salmonella* Typhimurium isolates from humans (n = 20) and foods (n = 20) in Brazil.

Research Institute	Isolate name	CFSAN nº	GenBank nº	Source	Year of isolation	Sequence Type (ST)
IAL-RP	STm02	CFSAN033849	LVHB00000000	Human feces	1983	19
IAL-RP	STm06	CFSAN033853	LVGX00000000	Human feces	1983	1649
IAL-RP	STm11	CFSAN033858	LVGT00000000	Human feces	1984	19
IAL-RP	STm17	CFSAN033864	LVGO00000000	Human feces	1985	19
IAL-RP	STm23	CFSAN033870	LVGJ00000000	Human feces	1986	19
IAL-RP	STm27	CFSAN033874	LVGF00000000	Human feces	1986	19
IAL-RP	STm28	CFSAN033875	LUJE00000000	Human feces	1988	3343
IAL-RP	STm29	CFSAN033876	LVGE00000000	Human feces	1989	313
IAL-RP	STm30	CFSAN033877	LVGD00000000	Human feces	1990	313
IAL-RP	STm31	CFSAN033878	LUJD00000000	Human feces	1991	19
IAL-RP	STm33	CFSAN033880	LVGB00000000	Human feces	1992	19
IAL-RP	STm34	CFSAN033881	LVGA00000000	Human feces	1993	313
IAL-RP	STm35	CFSAN033882	LVFZ00000000	Human feces	1995	313
IAL-RP	STm36	CFSAN033883	LVFY00000000	Cold chicken	1995	19
IAL-RP	STm37	CFSAN033884	LVFX00000000	Raw pork sausage	1996	313
IAL-RP	STm38	CFSAN033885	LUJC00000000	Human feces	1997	19
IAL-RP	STm39	CFSAN033886	LUJB00000000	Human feces	1998	313
IAL-RP	STm40	CFSAN033887	LUJA00000000	Lettuce	1998	313
IAL-RP	STm41	CFSAN033888	LVFW00000000	Raw kafta	1998	19
IAL-RP	STm42	CFSAN033889	LUIZ00000000	Human feces	1999	19
IAL-RP	STm44	CFSAN033891	LVFU00000000	Blood	2000	313
IAL-RP	STm45	CFSAN033892	LUIY00000000	Raw pork sausage	2000	19
IAL-RP	STm46	CFSAN033893	LVFT00000000	Raw tuscan sausage	2002	19
IAL-RP	STm47	CFSAN033894	LUIX00000000	Human feces	2003	313
IAL-RP	STm48	CFSAN033895	LUIW00000000	Brain abscess	2005	19
IAL-RP	STm49	CFSAN033896	LVFS00000000	Human feces	2010	19
FIOCRUZ	702/99	CFSAN033897	LVFR00000000	Final product	1999	19
FIOCRUZ	12278/06	CFSAN033899	LUIU00000000	Swine	2006	19
FIOCRUZ	5937/06	CFSAN033904	LUIQ00000000	Cold chicken	2006	19
FIOCRUZ	13609/06	CFSAN033909	LUIM00000000	Poultry	2006	19
FIOCRUZ	3848/08	CFSAN033910	LUIL00000000	Food	2008	19
FIOCRUZ	16238/09	CFSAN033911	LUIK00000000	Ready-to-eat dish	2009	19
FIOCRUZ	16273/09	CFSAN033916	LVFN00000000	Industrialized product	2009	19
FIOCRUZ	6346/10	CFSAN033922	LUIC00000000	Chicken	2010	19
FIOCRUZ	9109/10	CFSAN033924	LVFK00000000	Swine	2010	19
FIOCRUZ	6709/11	CFSAN033928	LVFJ00000000	Cold chicken	2011	19
FIOCRUZ	948/12	CFSAN033929	LUHY00000000	Raw salad	2012	19
FIOCRUZ	3330/12	CFSAN033932	LUHW00000000	Roast beef	2012	19
FIOCRUZ	583/13	CFSAN033938	LUHR00000000	Final product sales (animal origin)	2013	19
FIOCRUZ	623/13	CFSAN033939	LVFH00000000	Final product sales (animal origin)	2013	1921

### Whole genome sequencing

Whole genome sequencing of the 40 *S*. Typhimurium isolates (Table 1) was performed on the NextSeq platform (Illumina) at the U.S. Food and Drug Administration (FDA), College Park, MD, USA. The genomes were assembled using the software SPAdes [13] and the quality of the assemblies were evaluated using the software QUAST [14] as described in Almeida et al. [12].

### Invasion assay in Caco-2 epithelial cells and survival assay in U937 human macrophages

These assays were performed for all 40 *S*. Typhimurium isolates (Table 1) and for the SL1344 prototype strain according to [15–18].

Initially, the Caco-2 epithelial cells were cultured in DMEM medium (Dulbecco’s Modified Eagle Medium—Gibco—low glucose) supplemented with 10% fetal bovine serum (Life Technologies) and antibiotic in 5% CO_2_ at 37°C. In addition, 1x10^5^ cells were added to each well of a 12-well microplate. The assay was performed after 12 days of incubation until the cells were polarized and differentiated.

The monocytes were cultured in suspension in RPMI medium (1640—powder—Gibco) supplemented with 10% fetal bovine serum (Life Technologies) and antibiotic in 5% CO_2_ at 37°C. In addition, 1x10^5^ cells were added to each well of a 24-well microplate. For the differentiation of monocytes into macrophages, 1μl of phorbol 12-myristate-13-acetate (PMA) (Sigma-Aldrich) was used in 50 mL of the RPMI medium (50 ng/mL) and maintained in 5% CO_2_ at 37°C for 48h. All the *S*. Typhimurium strains were opsonized with 20% mouse serum (Sigma-Aldrich) at 37°C for 15 min after three washes with PBS (centrifuged at 12000 × rpm for 1 min).

The invasiveness in Caco-2 epithelial cells of these strains was performed after 90 minutes of bacteria-cell interaction with intraepithelial survival during 3 hours. In addition, the survival in U937 human macrophages was performed after 30 minutes of bacteria-cell interaction with intramacrophage survival during 3 hours and in both assays the (multiplicity of infection) MOI was 100:1.

Serial dilutions were performed and plated in LB agar medium plates with incubation during 18–24 hours at 37°C for later counting of colony forming units (CFU). The experiments were carried out in biological triplicate and in all plates there was a negative control with only cells in the wells.

### Virulence analysis in *Galleria mellonella*

The analysis it was performed according to [19]—adapted in *Galleria mellonella*. The larvae were maintained at 28°C in the dark in glass containers (30 cm height—20 cm wide—2 L capacity) with appropriate oxygen and access to food until reaching the sixth instar, whose weight is between 200 mg and 250 mg. After complete development, the larvae were deprived of food and separated into groups of 10 units in glass Petri dishes for each bacterial isolate and controls.

A Hamilton micro-syringe (model 7000.5KH of 10 μL) was used for artificial inoculation of *G*. *mellonella* larvae into the center of the last right pro-leg with 5 μL of *S*. Typhimurium (10^5^ CFU/mL) for each of the 40 isolates studied and for the positive control infected with *S*. Typhimurium strain ATCC14028. The negative control was inoculated with PBS. After inoculation, the larvae were incubated at 37°C, deprived of food and direct light. During the experimental period of 7 days the larvae were removed every 24 hours of the pre-pupae, in order to delay their metamorphosis with data recorded daily.

### Virulence gene characterization

The virulence genes were identified for all isolates using the Virulence Factors Database (VFDB) (http://www.mgc.ac.cn/VFs/main.htm). Bacteria *Salmonella* was assessedwith BLAST using a threshold of ≥70% identity, and ≥70% coverage comparing with *S*. *enterica* subsp. *enterica* serovar Typhimurium str. LT2, 4857432 bp, NC_003197, and plasmid *S*. *enterica* subsp. *enterica* serovar Typhimurium str. LT2 pSLT, 93939 bp, NC_003277 [20].

The presence of plasmids were determined using PlasmidFinder (Center for Genomic Epidemiology, https://cge.cbs.dtu.dk/services/PlasmidFinder/) with a threshold set for a minimum of 95% identity, and minimum coverage of 60% [21]. BLASTn (https://blast.ncbi.nlm.nih.gov/Blast.cgi) was used to confirm the location, and percentages of identity, and coverage between the *S*. *enterica* subsp. *enterica* serovar Typhimurium str. LT2 pSLT, 93939 bp, NC_003277 reference sequences of the pSLT plasmid, and the *spvABCDR*, *pefABCD*, *rck* and *mig-5* genes of the positive and negative genomes for IncFIIs.

### Statistical analyses

The comparisons between the means of the virulence tests were performed using Student's t-test for two means and two-way analysis of variance (ANOVA) with *post hoc* Tukey test for more than two means in the Minitab® statistical software (version 18.1). The analysis of the virulence genes identification was performed using two proportion z-test in the Minitab® statistical software (version 18.1) comparing the difference in isolates proportion which contained genes of interest. The graphics and statistical analyses of the virulence assay in *Galleria mellonella* were performed using the Log-rank (Mantel-Cox) method, both in the Prism5 program for Windows of the GraphPad® software (version 5.01). For all analyses, the level of significance was α = 5%.

## Results

### Caco-2 epithelial cells invasion assay

**Fig 1:**
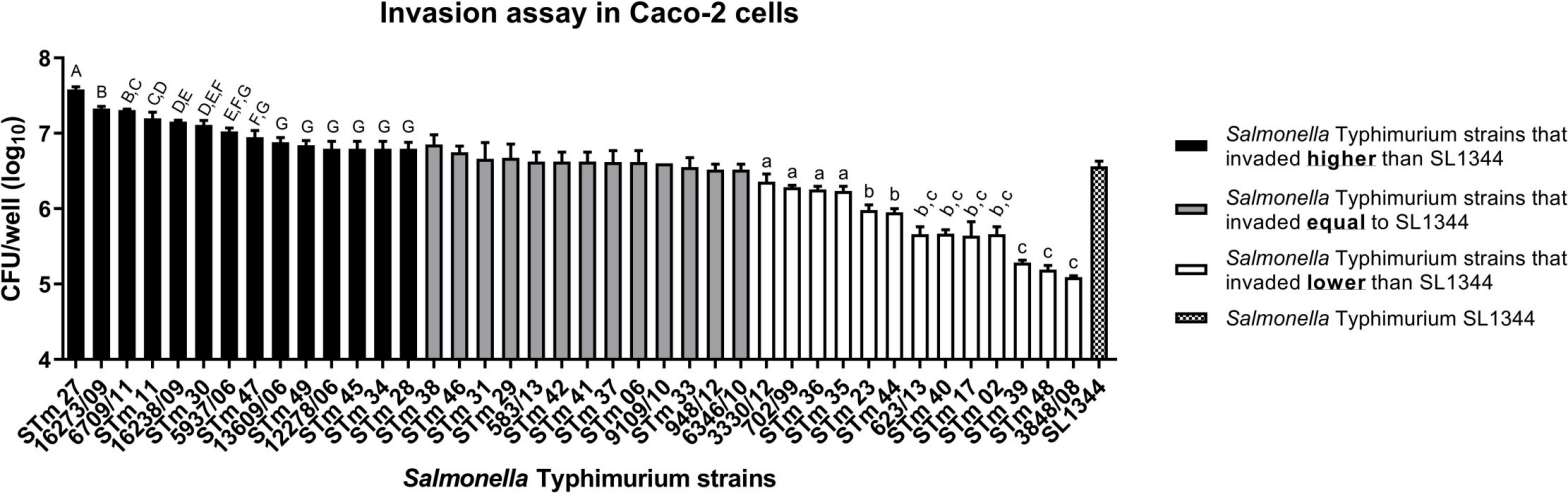
Invasion assay in Caco-2 epithelial cells for the 40 *S*. Typhimurium strains isolated from humans and foods and for the *S*. Typhimurium SL1344 reference. The means followed by different letters differ statistically from each other at a level of 5% significance. The error bars represent standard deviation of biological triplicate.

A total of 67.5% of the 40 *S*. Typhimurium isolates studied (32.5% isolated from humans and 35% isolated from food) invaded the epithelial cells at various levels compared to the SL1344 reference (Fig 1). Considering all the isolates studied the invasion in Caco-2 cells ranged from 1 × 10^5^ to 1 × 10^7^ CFU/mL. From the statistical analysis of the Student's t-test, three subgroups were found comprising: isolates that invaded more (black) (35%), equal (grey) (32.5%) or less (white) (32.5%) than the *S*. Typhimurium SL1344 isolate. The bidirectional analysis of variance (ANOVA) categorized the 14 *S*. Typhimurium isolates that invaded more than the SL1344 reference in groups from A to G. In addition, ANOVA also categorized the 13 *S*. Typhimurium isolates that invaded less than the SL1344 reference in groups from A to C (Fig 1).

### Survival assay in U937 human macrophages

**Fig 2:**
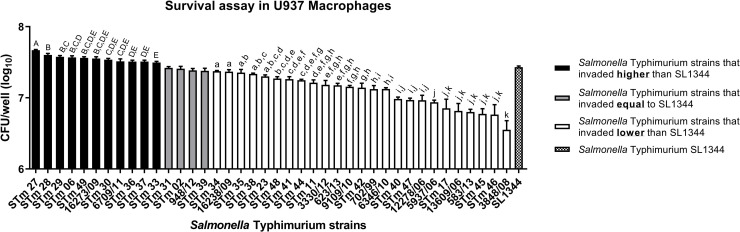
Survival assay in U937 human macrophages for the 40 *S*. Typhimurium strains isolated from humans and foods and for the *S*. Typhimurium SL1344 reference. The means followed by different letters differ statistically from each other at a level of 5% significance. The error bars represent standard deviation of biological triplicate.

A total of 37.5% of the 40 *S*. Typhimurium isolates studied (25% isolated from humans and 12.5% isolated from food) survived in the human macrophages at various levels compared to the SL1344 reference (Fig 2). All the isolates studied survived in U937 human macrophages and ranged from 1 × 10^6^ to 1 × 10^7^ CFU/mL. From the statistical analysis of Student's t-test, three subgroups were formed comprising: isolates that survived more (black) (27.5%), equal (grey) (10%) or less (white) (62.5%) than the *S*. Typhimurium SL1344 reference. The bidirectional analysis of variance (ANOVA) categorized the 11 *S*. Typhimurium isolates that survived more than the SL1344 reference in groups from A to E. In addition, ANOVA also categorized the 25 *S*. Typhimurium isolates that survived less than the SL134 reference in groups from A to K (Fig 2).

### Comparison between sources after invasion assay in Caco-2 epithelial cells and survival assay in U937 human macrophages

**Fig 3:**
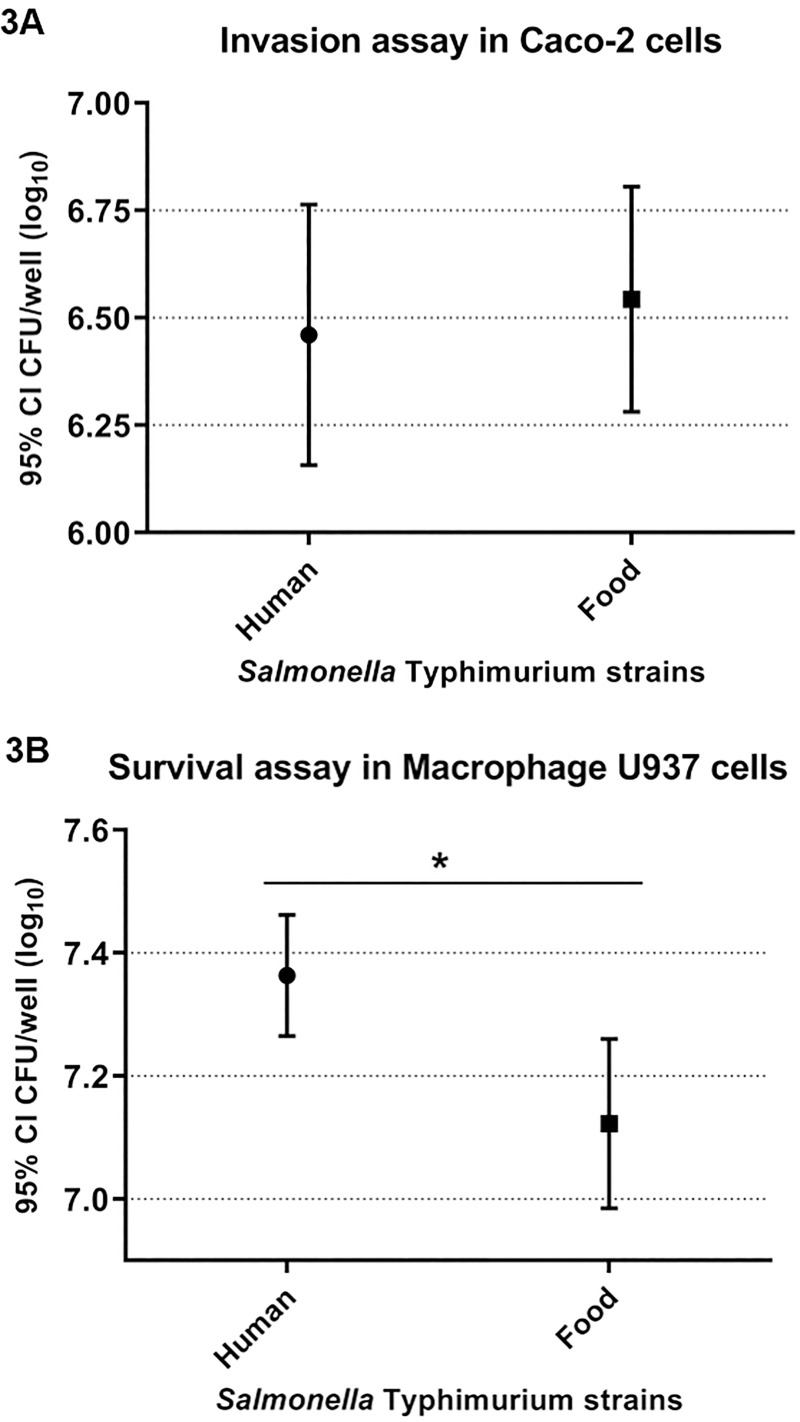
Comparison among the means of CFU/well (log_10_) with 95% confidence interval (CI) of *Salmonella* Typhimurium strains isolated from humans and foods after the assays of Caco-2 epithelial cell invasion (A) and U937 human macrophages survival (B).

In both assays, *S*. Typhimurium isolates were classified with invasion and survival levels similar, greater or lower than the SL1344 reference with the mean of CFU/well (Log_10_). Invasion in Caco-2 cells of 6.46 and 6.54 CFU/well (Log_10_) were observed for isolates from humans and foods, respectively (Fig 3A). Furthermore, the survival in U937 human macrophages was 7.36 and 7.12 CFU/well (Log_10_) for humans and food isolates, respectively (Fig 3B). Therefore, no evidence of statistically significant differences were found between isolates from humans and foods for invasion in Caco-2 cells. However, statistically significant differences were found between isolates from humans and foods for survival in U937 human macrophages.

### Virulence analysis in *Galleria mellonella*

**Fig 4:**
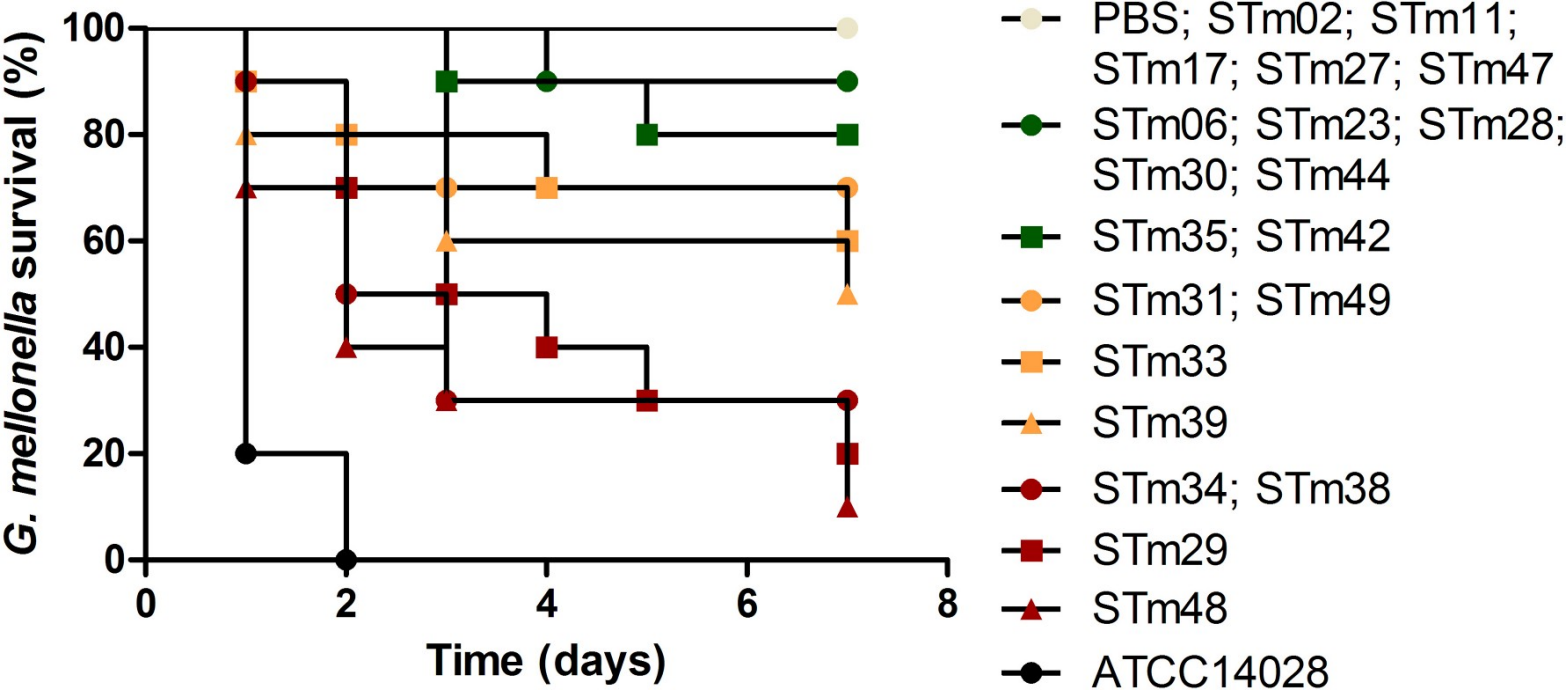
Survival percentages of *Galleria mellonella* larvae infected with 20 *Salmonella* Typhimurium isolates from humans in Brazil after seven days.

**Fig 5:**
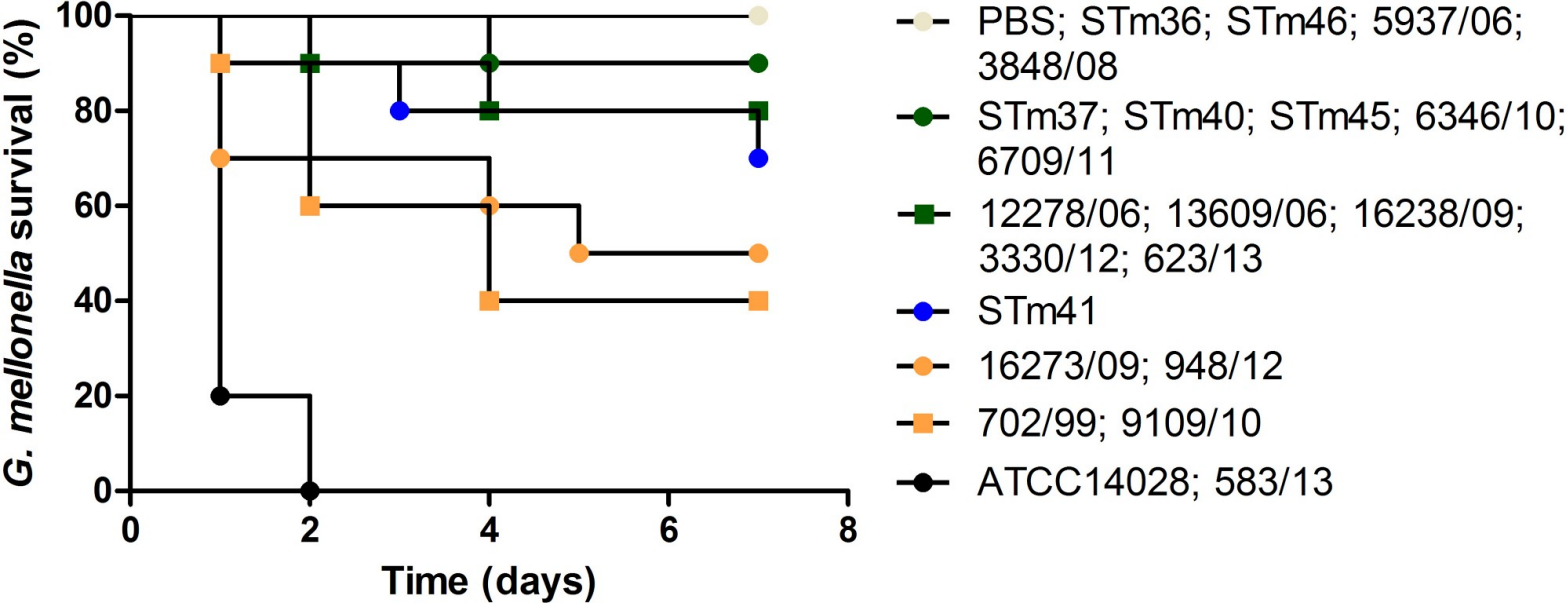
Survival percentages of *Galleria mellonella* larvae infected with 20 *Salmonella* Typhimurium isolates from foods in Brazil after seven days.

The results of the *S*. Typhimurium studied isolates are presented separately in Figs 4 and 5, respectively. In Fig 4, the ATCC14028 control strain isolated from chickens showed high virulence (black circle) killing 100% of the larvae. For human isolates, four groups were formed after seven days of experiments. The STm48, STm29, STm34 and STm38 isolates killed 70–90% of the larvae, forming the group of virulent isolates (red shapes). The intermediate virulence group (orange shapes) killed between 30–50% of the larvae, comprised of STm39, STm33, STm31 and STm49 isolates. The STm35, STm42, STm06, STm23, ST28, STm30 and STm44 isolates formed the group of low virulence (green shapes) killing between 10–20% of the larvae. Finally, the avirulent group (white circle) comprised of STm02, STm11, STm17, STm27, STm47 isolates and the negative control did not kill any larvae (Fig 4).

In Fig 5, four groups were formed after seven days of experiment for isolates from food. The 583/13 isolate and the positive control ATCC14028 showed a high virulence killing 100% of the larvae (black circle). The 702/99, 9109/10, 16273/09 and 948/12 isolates killed between 50–60% of the larvae, forming a group of intermediate virulence (orange shapes). The 12278/06, 13609/06, 16238/09, 3330/12, 623/13, STm37, STm40, STm45, 6346/10 and 6709/11 isolates formed a group of low virulence (green shapes) killing 10–20% of the larvae. Finally, the avirulent group (white circle) comprised of isolates STm36, STm46, 5937/06, 3848/08 and the negative control did not kill any larvae. Thus, there were fewer virulent isolates identified from foods compared to humans (Figs 4 and 5). Food isolate 583/13 was highly virulent and more so than any strain isolated from humans studied here in (Fig 5).

The STm41 isolate was not classified into either the intermediate or low virulence groups because its measurement fell between the two groups (Fig 5).

Comparing the mortality of the isolates belonging to the same group (human and food) no significant difference was observed. On the other hand, significant difference was found among the different groups mentioned above, except for the groups of low virulence (green shapes) and avirulent (white circle). These evidences were found by comparing representatives of each group.

### Genetic characterization focused on virulence genes

Genetic characterization focused on discovering virulence genes using the Virulence Factors Database (VFDB) which detected 153 genes related to invasion, survival, colonization, fimbriae and flagella production, among others for all isolates studied (Table 2).

**Table 2:** Proportion of the detection of virulence genes in *Salmonella* Typhimurium strains isolated from humans (n = 20) and food (n = 20) in Brazil.

Gene	Proportion of isolates	Query cover (%)	Identity (%)	Gene	Proportion of isolates	Query cover (%)	Identity (%)	Gene	Proportion of isolates	Query cover (%)	Identity (%)	Gene	Proportion of isolates	Query cover (%)	Identity (%)
*csgA*	40/40	100	100	*hilD*	39/40	100	99–100	*sprB*	40/40	100	100	*sseD*	40/40	100	99–100
*csgB*	40/40	100	100	*iagB*	40/40	100	100	*sptP*	40/40	98–100	99–100	*sseE*	40/40	100	99–100
*csgC*	40/40	100	100	*iacP*	40/40	100	100	**SPI-2**				*sseF*	40/40	100	100
*csgE*	40/40	100	99–100	*invA*	40/40	100	100	*orf32*	40/40	100	100	*sseG*	40/40	100	100
*csgF*	40/40	100	100	*invB*	40/40	100	100	*orf48*	40/40	100	100	*sseJ*	40/40	100	100
*csgG*	40/40	100	99–100	*invC*	40/40	100	100	*orf70*	40/40	100	98–100	*sspH2*	39/40	100	87–100
*fimA*	40/40	100	100	*invE*	40/40	100	100	*orf242*	40/40	100	99–100	*ssrA*	40/40	100	99–100
*fimC*	40/40	97–100	100	*invF*	40/40	86	100	*orf245*	40/40	100	100	*ssrB*	40/40	100	100
*fimD*	40/40	100	100	*invG*	40/40	100	100	*orf319*	40/40	100	100	*ttrA*	39/40	100	99–100
*fimF*	40/40	100	100	*invH*	40/40	100	100	*orf408*	40/40	100	100	*ttrB*	40/40	100	100
*fimH*	40/40	100	99–100	*invI*	40/40	100	100	*pykF*	40/40	100	100	*ttrC*	40/40	100	99–100
*fimI*	40/40	100	100	*invJ*	40/40	100	100	*sifA*	40/40	100	100	*ttrR*	40/40	100	100
*fimW*	40/40	100	100	*orgB*	40/40	100	99–100	*spiC/ssaB*	40/40	100	99–100	*ttrS*	40/40	100	99–100
*fimY*	40/40	100	100	*prgH*	40/40	95	100	*ssaC*	40/40	100	100	**SPI-3**			
*fimZ*	40/40	94–100	99–100	*prgI*	40/40	100	100	*ssaD*	38/40	100	99–100	*cigR*	39/40	90	99–100
*fur*	40/40	100	100	*prgJ*	40/40	100	99–100	*ssaE*	40/40	100	100	*fidL*	40/40	96–100	100
*lpfA*	40/40	100	100	*prgK*	40/40	100	99–100	*ssaG*	40/40	100	100	*marT*	40/40	100	100
												*mgtB mgtC misL*	40/40 40/40 40/40	100 100 s100	100 100 96–100
*lpfB*	40/40	100	99–100	*sicA*	40/40	100	100	*ssaH*	40/40	78	100	*slsA*	40/40	99–100	100
*lpfC*	39/40	100	99–100	*sicP*	40/40	99–100	10	*ssaI*	40/40	94	98–100	*sugR*	40/40	100	100
*lpfD*	40/40	100	100	*sipA/sspA*	40/40	100	99–100	*ssaJ*	40/40	100	100	*rhuM*	40/40	100	100
*lpfE*	40/40	100	100	*sipB/sspB*	40/40	100	99–100	*ssaK*	40/40	100	100	*rmbA*	40/40	100	100
				*sipC/sspC*	40/40	100	100	*ssaL*	40/40	100	100	**SPI-4**			
				*sipD*	40/40	100	100	*ssaM*	40/40	100	99–100	*siiE*	16/40	100	99–100
*mig-14*	40/40	96–100	100	*sitA*	39/40	100	100	*ssaN*	40/40	100	99–100	*soxR*	40/40	100	100
				*sitB*	39/40	100	100	*ssaO*	40/40	100	99–100	*soxS*	40/40	100	100
*phoP*	40/40	100	99–100	*sitC*	39/40	100	100	*ssaP*	40/40	100	99–100	*ssb*	40/40	100	100
*phoQ*	40/40	100	99–100	*sitD*	39/40	100	100	*ssaQ*	40/40	100	100	*yjcB*	40/40	100	100
*ratB*	40/40	98–100	99–100	*slrP*	40/40	100	99–100	*ssaR*	40/40	100	100	*yjcC*	40/40	100	100
*rpoS*	40/40	96–100	100	*sopA*	40/40	100	99–100	*ssaS*	40/40	100	100	**SPI-5**			
*shdA*	12/40	99	88–90	*sopB/sigD*	40/40	99–100	100	*ssaT*	40/40	100	100	*copR*	40/40	100	100
*sinH*	40/40	100	100	*sopD*	40/40	88	100	*ssaU*	40/40	100	99–100	*copS*	40/40	100	100
*sodCI*	40/40	100	100	*sopE2*	40/40	88–100	100	*ssaV*	40/40	100	100	*orfX*	31/40	100	97
**SPI-1**				*spaO*	40/40	100	100	*sscA*	40/40	100	99–100	*pipA*	40/40	97–100	100
*avrA*	38/40	90–100	95–100	*spaP*	40/40	100	99–100	*sscB*	40/40	100	99–100	*pipB*	40/40	100	100
*fhlA*	39/40	93–100	99–100	*spaQ*	40/40	100	100	*sseA*	40/40	100	100	*pipC*	40/40	88–100	100
*hilA*	40/40	100	100	*spaR*	40/40	100	100	*sseB*	40/40	100	99–100	*pipD*	40/40	83–94	100
*hilC*	39/40	100	99–100	*spaS*	40/40	100	99–100	*sseC*	40/40	100	99–100				
*csgA*	40/40	100	100	*invA*	40/40	100	100	*orf32*	40/40	100	100	*sseG*	40/40	100	100
*csgB*	40/40	100	100	*invB*	40/40	100	100	*orf48*	40/40	100	100	*sseJ*	40/40	100	100
*csgC*	40/40	100	100	*invC*	40/40	100	100	*orf70*	40/40	100	98–100	*sspH2*	39/40	100	87–100
*csgE*	40/40	100	99–100	*invE*	40/40	100	100	*orf242*	40/40	100	99–100	*ssrA*	40/40	100	99–100
*csgF*	40/40	100	100	*invF*	40/40	86	100	*orf245*	40/40	100	100	*ssrB*	40/40	100	100
*csgG*	40/40	100	99–100	*invG*	40/40	100	100	*orf319*	40/40	100	100	*ttrA*	39/40	100	99–100
*fimA*	40/40	100	100	*invH*	40/40	100	100	*orf408*	40/40	100	100	*ttrB*	40/40	100	100
*fimC*	40/40	97–100	100	*invI*	40/40	100	100	*pykF*	40/40	100	100	*ttrC*	40/40	100	99–100
*fimD*	40/40	100	100	*invJ*	40/40	100	100	*sifA*	40/40	100	100	*ttrR*	40/40	100	100
*fimF*	40/40	100	100	*orgB*	40/40	100	99–100	*spiC/ssaB*	40/40	100	99–100	*ttrS*	40/40	100	99–100
*fimH*	40/40	100	99–100	*prgH*	40/40	95	100	*ssaC*	40/40	100	100	**SPI-3**			
*fimI*	40/40	100	100	*prgI*	40/40	100	100	*ssaD*	38/40	100	99–100	*cigR*	39/40	90	99–100
*fimW*	40/40	100	100	*prgJ*	40/40	100	99–100	*ssaE*	40/40	100	100	*fidL*	40/40	96–100	100
*fimY*	40/40	100	100	*prgK*	40/40	100	99–100	*ssaG*	40/40	100	100	*marT*	40/40	100	100
*fimZ*	40/40	94–100	99–100	*sicA*	40/40	100	100	*ssaH*	40/40	78	100	*mgtB*	40/40	100	100
*Fur*	40/40	100	100	*sicP*	40/40	99–100	10	*ssaI*	40/40	94	98–100	*mgtC*	40/40	100	100
*lpfA*	40/40	100	100	*sipA/sspA*	40/40	100	99–100	*ssaJ*	40/40	100	100	*misL*	40/40	100	100; 96
*lpfB*	40/40	100	99–100	*sipB/sspB*	40/40	100	99–100	*ssaK*	40/40	100	100	*slsA*	40/40	99–100	100
*lpfC*	39/40	100	99–100	*sipC/sspC*	40/40	100	100	*ssaL*	40/40	100	100	*sugR*	40/40	100	100
*lpfD*	40/40	100	100	*sipD*	40/40	100	100	*ssaM*	40/40	100	99–100	*rhuM*	40/40	100	100
*lpfE*	40/40	100	100	*sitA*	39/40	100	100	*ssaN*	40/40	100	99–100	*rmbA*	40/40	100	100
*mig-14*	40/40	96–100	100	*sitB*	39/40	100	100	*ssaO*	40/40	100	99–100	**SPI-4**			
*phoP*	40/40	100	99–100	*sitC*	39/40	100	100	*ssaP*	40/40	100	99–100	*siiE*	16/40	100	99–100
*phoQ*	40/40	100	99–100	*sitD*	39/40	100	100	*ssaQ*	40/40	100	100	*soxR*	40/40	100	100
*ratB*	40/40	98–100	99–100	*slrP*	40/40	100	99–100	*ssaR*	40/40	100	100	*soxS*	40/40	100	100
*rpoS*	40/40	96–100	100	*sopA*	40/40	100	99–100	*ssaS*	40/40	100	100	*ssb*	40/40	100	100
*shdA*	12/40	99	88–90	*sopB/sigD*	40/40	99–100	100	*ssaT*	40/40	100	100	*yjcB*	40/40	100	100
*sinH*	40/40	100	100	*sopD*	40/40	88	100	*ssaU*	40/40	100	99–100	*yjcC*	40/40	100	100
*sodCI*	40/40	100	100	*sopE2*	40/40	88–100	100	*ssaV*	40/40	100	100	**SPI-5**			
**SPI-1**	*spaO*	40/40	100	100	*sscA*	40/40	100	99–100	*copR*	40/40	100	100			
*avrA*	38/40	90–100	95–100	*spaP*	40/40	100	99–100	*sscB*	40/40	100	99–100	*copS*	40/40	100	100
*fhlA*	39/40	93–100	99–100	*spaQ*	40/40	100	100	*sseA*	40/40	100	100	*orfX*	31/40	100	97
*hilA*	40/40	100	100	*spaR*	40/40	100	100	*sseB*	40/40	100	99–100	*pipA*	40/40	97–100	100
*hilC*	39/40	100	99–100	*spaS*	40/40	100	99–100	*sseC*	40/40	100	99–100	*pipB*	40/40	100	100
*hilD*	39/40	100	99–100	*sprB*	40/40	100	100	*sseD*	40/40	100	99–100	*pipC*	40/40	88–100	100
*iagB*	40/40	100	100	*sptP*	40/40	98–100	99–100	*sseE*	40/40	100	99–100	*pipD*	40/40	83–94	100
*iacP*	40/40	100	100	**SPI-2**	*sseF*	40/40	100	100							

The similarity rate varied between 87% and 100% of identity for all strains with coverage between 78% and 100% (Table 2). The *spvABCDR* operon, *pefABCD* operon, *rck* and *mig-5* genes were detected in 13 *S*. Typhimurium isolates from humans and 9 isolates from foods (Table 3). In order to confirm the location of genes, Plasmid Finder was used to document the presence of the IncFIIs plasmid incompatibility group, which belongs to the pSLT plasmid (Table 4). Independent, BLASTn analysis for all isolates confirmed the presence of the pSLT plasmid carrying the *spvABCDR* operon, *pefABCD* operon, *rck* and *mig-5* genes for 22 *S*. Typhimurium isolates with coverage of 100% and 99–100% of identity (Table 4). The other studied isolates did not present IncFIIs, pSLT plasmid, *spvABCDR* operon, *pefABCD* operon, *rck* and *mig-5* genes.

**Table 3:** Characteristics of the plasmid genes presented in 22 *Salmonella* Typhimurium isolates by Virulence factors database (VFDB).

Gene	Human			Food		
	Proportion of isolates	Query cover (%)	Identity (%)	Proportion of isolates	Query cover (%)	Identity (%)
***spvA***	13/22	100	100	9/22	100	100
***spvB***	13/22	100	100	9/22	100	100
***spvC***	13/22	100	100	9/22	100	100
***spvD***	13/22	100	99–100	9/22	100	100
***spvR***	13/22	100	100	9/22	100	100
***pefA***	13/22	100	100	9/22	100	100
***pefB***	13/22	100	100	9/22	100	100
***pefC***	13/22	100	99–100	9/22	100	99–100
***pefD***	13/22	100	100	9/22	100	100
***rck***	13/22	100	100	9/22	100	100
***mig-5***	13/22	100	100	9/22	100	100

**Table 4:** PlasmidFinder and BLASTn of 22 *Salmonella* Typhimurium isolates that presented the pSLT plasmid carrying the plasmid genes studied.

Isolate name	CFSAN nº	IncF plasmid (Identity %)	pSLT (Query cover %) (Identity %)	*spvABCDR* (Query cover %) (Identity %)	*pefABCD* (Query cover %) (Identity %)	*mig-5* (Query cover %) (Identity %)
STm06	CFSAN033853	IncFIIs (100)	(99) (99.97)	(100) (100)	(100) (99.74)	(100) (99.70)
STm11	CFSAN033858	IncFIIs (100)	(99) (99.91)	(100) (100)	(100) (99.81)	(100) (99.70)
STm27	CFSAN033874	IncFIIs (100)	(99) (99.90)	(100) (100)	(100) (99.81)	(100) (99.70)
STm29	CFSAN033876	IncFIIs (100)	(99) (99.92)	(100) (100)	(100) (99.77)	(100) (99.70)
STm30	CFSAN033877	IncFIIs (100)	(99) (99.93)	(100) (100)	(100) (99.77)	(100) (99.70)
STm31	CFSAN033878	IncFIIs (100)	(99) (99.94)	(100) (99.94)	(100) (99.81)	(100) (99.70)
STm33	CFSAN033880	IncFIIs (100)	(99) (99.93)	(100) (99.94)	(100) (99.81)	(100) (99.70)
STm34	CFSAN033881	IncFIIs (100)	(99) (99.93)	(100) (100)	(100) (99.77)	(100) (99.70)
STm35	CFSAN033882	IncFIIs (100)	(99) (99.93)	(100) (100)	(100) (99.77)	(100) (99.70)
STm36	CFSAN033883	IncFIIs (100)	(99) (99.92)	(100) (99.66)	(100) (99.81)	(100) (99.70)
STm37	CFSAN033884	IncFIIs (100)	(99) (99.94)	(100) (100)	(100) (99.77)	(100) (99.70)
STm38	CFSAN033885	IncFIIs (100)	(99) (99.98)	(100) (100)	(100) (99.71)	(100) (99.70)
STm39	CFSAN033886	IncFIIs (100)	(99) (99.93)	(100) (100)	(100) (99.77)	(100) (99.70)
STm40	CFSAN033887	IncFIIs (100)	(99) (99.92)	(100) (99.94)	(100) (99.77)	(100) (99.70)
STm44	CFSAN033891	IncFIIs (100)	(99) (99.94)	(100) (100)	(100) (99.77)	(100) (99.70)
STm47	CFSAN033894	IncFIIs (100)	(99) (99.94)	(100) (100)	(100) (99.77)	(100) (99.70)
13609/06	CFSAN033909	IncFIIs (100)	(99) (99.96)	(100) (100)	(100) (99.74)	(100) (99.56)
16273/09	CFSAN033916	IncFIIs (100)	(99) (99.96)	(100) (100)	(100) (99.71)	(100) (99.74)
6346/10	CFSAN033922	IncFIIs (100)	(99) (99.90)	(100) (100)	(100) (99.77)	(100) (99.65)
9109/10	CFSAN033924	IncFIIs (100)	(99) (99.96)	(100) (100)	(100) (99.74)	(100) (99.70)
948/12	CFSAN033929	IncFIIs (100)	(99) (99.97)	(100) (100)	(100) (99.74)	(100) (99.65)
3330/12	CFSAN033932	IncFIIs (100)	(99) (99.95)	(100) (99.94)	(100) (99.74)	(100) (99.61)

The *rck* gene was detected for all isolates with query cover and identity of 100%.

## Discussion

In this study, 40 S. Typhimurium isolates from humans (n = 20) and foods (n = 20) in Brazil were compared after invasion assays in Caco-2 epithelial cells, survival assays in U937 human macrophages, *Galleria mellonella* assays and virulence gene analysis.

*S*. Typhimurium being considered a generalist serovar has been documented to infect several hosts including humans, cattle, pigs, sheep, horses, rodents, chickens, turkeys, ducks, pigeons, and birds [22, 23]. This serovar invades host cells through the Type III Secretion System (T3SS) where the genes are mainly located in pathogenicity island 1 and 2 [24–26].

Among the 40 *S*. Typhimurium isolates studied, from humans and foods many invaded the epithelial cells at similar or higher levels compared to the SL1344 reference control. Furthermore, analysis of variance (ANOVA) was performed for all *S*. Typhimurium isolates that differed from the SL1344 reference (Fig 1). By this analysis three main groups were formed and the source and/or year of isolation did not correlate with the observed virulence profiles. Therefore, these results reinforce that the ability for *S*. Typhimurium to invade is probably isolate dependent and not related to the source or the year of isolation (Fig 3A).

During the infection process, host neutrophils and macrophages try to control invasion by generating reactive oxygen species (ROS). The degranulation of these cells occurs in a process called respiratory burst [27].

*S*. Typhimurium has the ability to infect epithelial cells and macrophages in the small intestine, replicating in a niche called *Salmonella*-containing vacuole (SCV) and consequently triggering an inflammatory process culminating in gastroenteritis [28]. It is important to emphasize that in the present study, among the 40 *S*. Typhimurium strains studied, from humans and food survived at various levels compared to SL1344 control. In addition, analysis of variance (ANOVA) was performed for all *S*. Typhimurium strains that differed from the SL1344 control (Fig 2). Three main groups were formed by this analysis and statistical significance was observed among the profiles, suggesting that *S*. Typhimurium isolates from humans survive more intramacrophage than isolates from food (Fig 3B).

Among the possible causes of this difference we highlight a lesser dispersion of the survival assay data in U937 human macrophages and subtle differences in the genetic characterization for some important virulence genes.

The virulence assay in *Galleria mellonella* larvae divided the 40 *S*. Typhimurium isolates into four groups according to their virulence profiles (Figs 4 and 5). It is important to emphasize that there was a higher proportion (60%) of strains isolated from humans that demonstrated a virulent profile in comparison to strains isolated from foods. Therefore, this result may suggest that the *S*. Typhimurium studied isolated from humans were more virulent than strains isolated from food in Brazil according to the *G*. *mellonella* infection model.

*G*. *mellonella* larvae are easily grown in large numbers at low costs and produce innate immune response components very similar to humans composed by hemocytes and opsonins [9]. A limitation of this infection model is that insects do not present the second line of defense characterized by an adaptive immune response formed by antibody-producing and memory cells [9].

It is important to mention that the melanization of insects such as *G*. *mellonella* occurs when it is infected by a pathogen, followed by melanin synthesis and deposition of this substance to encapsulate the infectious agents at the inoculation site [29]. Therefore, this process is stimulated by the presence of bacteria and fungi, initiating a serine protease cascade responsible for the activation of phenoloxidase that catalyzes the formation of melanin [9, 29].

The genetic repertoire research focused in virulence genes by WGS revealed that the isolates regardless of the source were very similar. Moreover, several essential genes for the pathogenesis of salmonellosis were identified in this study with high identity and coverage reinforcing the pathogenic potential of these strains. It is important to mention that the ability of *S*. Typhimurium strains to invade and to survive in the host cells is closely linked to virulence genes present in the bacterium [30].

The *invG*, *invH*, *prgH*, *prgK*, *prgI*, *prgJ* and *iagB* genes are found in the SPI-1 of *Salmonella* spp. and are involved with the formation of the basal body of the T3SS [31]. The export apparatus of the T3SS are encoded by the *spaS*, *spaP*, *spaQ*, *spaR* and *invA* genes present in the SPI-1. Furthermore, the *spaO*, *invC*, *invI*, *orgB*, *invJ*, *invE*, *sipC*, *sipB* and *sipD* genes also are present in the SPI-1 and related to cytoplasmic ring, ATPase complex, regulation and translocation of the T3SS [31].

According to Deng and collaborators (2017), the *ssaC*, *ssaD*, *ssaJ*, *ssaG*, *ssaI*, *ssaU*, *ssaV*, *ssaR*, *ssaS*, *ssaT*, *ssaQ*, *ssaN*, *ssaK*, *ssaO*, *ssaP*, *ssaL*, *spiC*, *sseD*, *sseC and sseB* genes also encode proteins related to basal body, export apparatus, cytoplasmic ring, ATPase complex, regulation and translocation of the T3SS and are located in the SPI-2 de *Salmonella* spp [31].

All the genes mentioned above were found in the *S*. Typhimurium genomes of this study, suggesting that although these strains belong to collections from different years the essential genes for the injection of T3SS effectors proteins have been preserved (Table 2).

Moreover, genes present in the SPI-3, SPI4 and SPI-5 are related to virulence of *Salmonella* spp. However, more studies are needed in this area, due to the lack of theoretical and scientific information [30]. For example, the *pip* (*pipA*, *pipB*, *pipC* and *pipD*) genes may be related to the rate of fluid secretion and inflammatory response during salmonellosis, suggesting that such genes are related to the bacterial enteropathogenicity, but the exact mechanism has not been yet fully elucidated [30, 32].

Plasmids are known to be essential for the resistance and virulence of different bacteria, the IncF plasmid incompatibility group is heterogeneous and often described in enterobacteria [33]. The virulence plasmid (pSLT) which belongs to the IncFIIs plasmid incompatibility group has been reported in *S*. Typhimurium and carries important genes for the pathogenesis of this serovar [34]. Among the content of the plasmid pSLT can highlight the following genes: *spvABCDR*, *pefABCD*, *rck* and *mig-5*.

The *spv* operon (*Salmonella* plasmid virulence) which is formed of five genes (*spvA*, *spvB*, *spvC*, *spvD* and *spvR*) has been associated with *Salmonella* spp. survival and multiplication in macrophages [35]. In addition, the *pef* fimbrial operon (plasmid encoded fimbriae) is responsible for the adhesion of *Salmonella* spp. to intestinal epithelium in infant mouse resulting in fluid accumulation and is consisted by four genes (*pefA*, *pefB*, *pefC* and *pefD*) [36, 37].

It is important to emphasize that the *rck* (resistance to complement killing) and *mig-5* (macrophage inducible gene coding for putative carbonic anhydrase) plasmid genes have been associated to the resistance of *S*. Typhimurium strains to the host complement system which would cause bacterial cell disruption and related to neutralization of toxic compounds produced by macrophages after phagocytosis, respectively [35].

Interestingly, plasmid genes (*spvABCDR*, *pefABCD*, *rck* and *mig-5*) were detected in the present work in equal numbers and its location was confirmed in the plasmid pSLT, being that the *S*. Typhimurium strains isolated from humans and food studied had all the plasmid genes mentioned previously or none (Tables 3 and 4). According to Kuijpers and collaborators (2019), the *spvABCDR*, *pefABCD*, *rck* and *mig-5* plasmids genes were detected only in the human cases associated with serovars Typhimurium and Enteritidis [38].

The predominant sequence type (ST) of *S*. Typhimurium from fecal samples has been ST19 worldwide. However, ST313 has caused a significant mortality rate in sub-Saharan Africa and has been described in recent years in Brazil [39]. In the present study, all the ST313 isolates presented the plasmid pSLT carrying the *spvABCDR* operon, *pefABCD* operon, *rck* and *mig-5* genes showing a high genomic similarity among each other regardless of the isolation source as previously described [40].

Considering that the *S*. Typhimurium strains isolated from humans survived more in U937 human macrophages than strains isolated from food in the present study, it is important to emphasize that a complex response like survival in human macrophages probably is not triggered for one or a few genes, highlighting specifically the importance of plasmidial gene groups. In addition, despite the high similarity of the genes present among the isolates studied, a difference was observed in the proportion of human isolates which contained the plasmid gene group *spvA*, *spvB*, *spvC*, *spvD*, *spvR*, *pefA*, *pefB*, *pefC*, *pefD*, *rck* and *mig-5* between *S*. Typhimurium strains isolated from humans and foods.

Therefore, a statistically significant difference was found (p-value = 0,001) suggesting that a higher proportion of human isolates which contained these genes may contribute to their greater survival in U937 human macrophages. It is important to mention that this plasmid gene group was the only one that showed the same pattern of presence/absence among the isolates. Furthermore, it was also the only group in which p-value differed statistically, calculated using both the normal approximation method and the Fisher's exact method, that latter tends to be more conservative, reinforcing the statistical significant difference.

Similarly, among 9 (69%) of the 13 *S*. Typhimurium isolates from humans and foods that had a virulent profile (high and intermediate virulence) in *G*. *mellonella*, the plasmid gene group was detected reinforcing their importance in the virulence of these isolates.

Finally, many other genes were detected in the *S*. Typhimurium genomes in the present study, specifically genes related to the fimbriae production, such as the *fim* (f*imA*, *fimC*, *fimD*, *fimF*, *fimH*, *fimI*, *fimW*, *fimY* and *fimZ*) genes, which encode structural subunits and fimbrial proteins. The *lpf* (*lpfA*, *lpfB*, *lpfC*, *lpfD* and *lpfE*) fimbrial genes have been described as important in the intestinal colonization in murine mucosa [36, 37]. Moreover, no relationship was found between the isolates proportion that presented these researched chromosomal genes suggesting that the different profiles found in the invasion assay in Caco-2 epithelial cells and in the survival assay in U937 human macrophages can be linked to the expression of such genes.

In conclusion, a significant percentage of *S*. Typhimurium isolates from humans and foods showed high invasion in Caco-2 epithelial cells regardless of the source suggesting that the invasiveness to Caco-2 cells is probably isolated dependent and not related to the source or the year of isolation. However, *S*. Typhimurium isolates from humans showed greater survival rate in U937 human macrophages and higher proportion of isolates with a virulent profile in *Galleria mellonella* than isolates from foods suggesting that this difference may be related to the higher proportion of human isolates which contained plasmid genes, such as *spvABCDR*, *pefABCD*, *rck* and *mig-5*. Moreover, several virulence genes present in the pathogenicity islands 1, 2, 3, 4, 5 were detected in the *S*. Typhimurium isolates from humans and foods studied reinforcing the virulence of this important serovar independent of their clinical or non-human origin.

Altogether, the results obtained in this work contributed for a better characterization of *S*. Typhimurium isolates from humans and foods in Brazil over decades regarding itsability to invade Caco-2 epithelial cells, to survive in U937 human macrophages, virulence, and pathogenic potential.
