# Correction: Phenotypic and genotypic characterization of *Salmonella* Typhimurium isolates from humans and foods in Brazil

**DOI:** 10.1371/journal.pone.0240055

**Published:** 2020-09-25

**Authors:** Amanda Aparecida Seribelli, Marcelo Ferreira Cruz, Felipe Pinheiro Vilela, Miliane Rodrigues Frazão, Mario H. Paziani, Fernanda Almeida, Marta Inês Cazentini Medeiros, Dália dos Prazeres Rodrigues, Marcia R. von Zeska Kress, Marc W. Allard, Juliana Pfrimer Falcão

There are errors in Table 2. Multiple rows in Table 2 are incorrectly duplicated. Please see the correct Table 2 here.

**Table 2:** Proportion of the detection of virulence genes in *Salmonella* Typhimurium strains isolated from humans (n = 20) and food (n = 20) in Brazil.

Gene	Proportion of isolates	Query cover (%)	Identity (%)	Gene	Proportion of isolates	Query cover (%)	Identity (%)	Gene	Proportion of isolates	Query cover (%)	Identity (%)	Gene	Proportion of isolates	Query cover (%)	Identity (%)
*csgA*	40/40	100	100	*invA*	40/40	100	100	*orf32*	40/40	100	100	*sseG*	40/40	100	100
*csgB*	40/40	100	100	*invB*	40/40	100	100	*orf48*	40/40	100	100	*sseJ*	40/40	100	100
*csgC*	40/40	100	100	*invC*	40/40	100	100	*orf70*	40/40	100	98–100	*sspH2*	39/40	100	87–100
*csgE*	40/40	100	99–100	*invE*	40/40	100	100	*orf242*	40/40	100	99–100	*ssrA*	40/40	100	99–100
*csgF*	40/40	100	100	*invF*	40/40	86	100	*orf245*	40/40	100	100	*ssrB*	40/40	100	100
*csgG*	40/40	100	99–100	*invG*	40/40	100	100	*orf319*	40/40	100	100	*ttrA*	39/40	100	99–100
*fimA*	40/40	100	100	*invH*	40/40	100	100	*orf408*	40/40	100	100	*ttrB*	40/40	100	100
*fimC*	40/40	97–100	100	*invI*	40/40	100	100	*pykF*	40/40	100	100	*ttrC*	40/40	100	99–100
*fimD*	40/40	100	100	*invJ*	40/40	100	100	*sifA*	40/40	100	100	*ttrR*	40/40	100	100
*fimF*	40/40	100	100	*orgB*	40/40	100	99–100	*spiC/ssaB*	40/40	100	99–100	*ttrS*	40/40	100	99–100
*fimH*	40/40	100	99–100	*prgH*	40/40	95	100	*ssaC*	40/40	100	100	**SPI-3**			
*fimI*	40/40	100	100	*prgI*	40/40	100	100	*ssaD*	38/40	100	99–100	*cigR*	39/40	90	99–100
*fimW*	40/40	100	100	*prgJ*	40/40	100	99–100	*ssaE*	40/40	100	100	*fidL*	40/40	96–100	100
*fimY*	40/40	100	100	*prgK*	40/40	100	99–100	*ssaG*	40/40	100	100	*marT*	40/40	100	100
*fimZ*	40/40	94–100	99–100	*sicA*	40/40	100	100	*ssaH*	40/40	78	100	*mgtB*	40/40	100	100
*fur*	40/40	100	100	*sicP*	40/40	99–100	10	*ssaI*	40/40	94	98–100	*mgtC*	40/40	100	100
*lpfA*	40/40	100	100	*sipA/sspA*	40/40	100	99–100	*ssaJ*	40/40	100	100	*misL*	40/40	100	96–100
*lpfB*	40/40	100	99–100	*sipB/sspB*	40/40	100	99–100	*ssaK*	40/40	100	100	*slsA*	40/40	99–100	100
*lpfC*	39/40	100	99–100	*sipC/sspC*	40/40	100	100	*ssaL*	40/40	100	100	*sugR*	40/40	100	100
*lpfD*	40/40	100	100	*sipD*	40/40	100	100	*ssaM*	40/40	100	99–100	*rhuM*	40/40	100	100
*lpfE*	40/40	100	100	*sitA*	39/40	100	100	*ssaN*	40/40	100	99–100	*rmbA*	40/40	100	100
*mig-14*	40/40	96–100	100	*sitB*	39/40	100	100	*ssaO*	40/40	100	99–100	**SPI-4**			
*phoP*	40/40	100	99–100	*sitC*	39/40	100	100	*ssaP*	40/40	100	99–100	*siiE*	16/40	100	99–100
*phoQ*	40/40	100	99–100	*sitD*	39/40	100	100	*ssaQ*	40/40	100	100	*soxR*	40/40	100	100
*ratB*	40/40	98–100	99–100	*slrP*	40/40	100	99–100	*ssaR*	40/40	100	100	*soxS*	40/40	100	100
*rpoS*	40/40	96–100	100	*sopA*	40/40	100	99–100	*ssaS*	40/40	100	100	*ssb*	40/40	100	100
*shdA*	12/40	99	88–90	*sopB/sigD*	40/40	99–100	100	*ssaT*	40/40	100	100	*yjcB*	40/40	100	100
*sinH*	40/40	100	100	*sopD*	40/40	88	100	*ssaU*	40/40	100	99–100	*yjcC*	40/40	100	100
*sodCI*	40/40	100	100	*sopE2*	40/40	88–100	100	*ssaV*	40/40	100	100	**SPI-5**			
**SPI-1**				*spaO*	40/40	100	100	*sscA*	40/40	100	99–100	*copR*	40/40	100	100
*avrA*	38/40	90–100	95–100	*spaP*	40/40	100	99–100	*sscB*	40/40	100	99–100	*copS*	40/40	100	100
*fhlA*	39/40	93–100	99–100	*spaQ*	40/40	100	100	*sseA*	40/40	100	100	*orfX*	31/40	100	97
*hilA*	40/40	100	100	*spaR*	40/40	100	100	*sseB*	40/40	100	99–100	*pipA*	40/40	97–100	100
*hilC*	39/40	100	99–100	*spaS*	40/40	100	99–100	*sseC*	40/40	100	99–100	*pipB*	40/40	100	100
*hilD*	39/40	100	99–100	*sprB*	40/40	100	100	*sseD*	40/40	100	99–100	*pipC*	40/40	88–100	100
*iagB*	40/40	100	100	*sptP*	40/40	98–100	99–100	*sseE*	40/40	100	99–100	*pipD*	40/40	83–94	100
*iacP*	40/40	100	100	**SPI-2**				*sseF*	40/40	100	100				
